# Reassortment of Ancient Neuraminidase and Recent Hemagglutinin in Pandemic (H1N1) 2009 Virus

**DOI:** 10.3201/eid1611.100361

**Published:** 2010-11

**Authors:** Priyasma Bhoumik, Austin L. Hughes

**Affiliations:** Author affiliation: University of South Carolina, Columbia, South Carolina, USA

**Keywords:** Pandemic (H1N1) 2009 virus, influenza A virus, reassortment, sequence polymorphism, viral evolution, viruses, neuraminidase, hemagglutinin, dispatch

## Abstract

Sequence analyses show that the outbreak of pandemic (H1N1) 2009 resulted from the spread of a recently derived hemagglutinin through a population of ancient and more diverse neuraminidase segments. This pattern implies reassortment and suggests that the novel form of hemagglutinin conferred a selective advantage.

## Correction

*In this article, errors were made in selection of the hemagglutinin (HA) and neuraminidase (NA) sequences for the initial and subsequent data sets. As a result, the authors incorrectly concluded that the NA gene of the pandemic (H1N1) 2009 virus is of a more ancient lineage than the HA. Other researchers (and the authors) have not been able to reproduce the findings when using HA and NA matched pairs from viruses chosen on the basis of geography and time and correctly have pointed out errors in the data set that make the original conclusions invalid.*

Influenza virus A is a single-strand, negative-sense RNA virus whose genome consists of 8 RNA segments that encode 10 proteins (*1*). Influenza A is endemic in wild waterfowl, from which new strains periodically emerge to infect mammals, including humans and domestic pigs (*2*). Strains of influenza A viruses are categorized according to serotypes for hemagglutinin (HA) and neuraminidase (NA) proteins. These proteins cover the surface of the virus, are the main targets of the host’s cellular immune response, and play major roles in the infection process (*1**,**3**,**4*).

In 2009, a novel strain of influenza A virus, pandemic (H1N1) 2009 virus, appeared in the human population, infecting thousands and causing many deaths (*2**,**5**–**8*). Phylogenetic analyses support a close relationship between the new strain and the strains that infect swine (*6**–**9*). Because different segments of the pandemic (H1N1) 2009 virus genome show different patterns of relationship to previously identified clades of influenza A virus sequences, these analyses support a role for intersegment reassortment in the origin of the new strain (*6**–**9*). For example, HA of pandemic (H1N1) 2009 virus shows a close relationship to that of classical swine influenza A virus, and NA shows a close relationship to that of Eurasian swine influenza A virus (*6**–**9*).

## The Study

To examine the effects of intersegment reassortment on sequence diversity, we analyzed the pattern of nucleotide substitutions in pandemic (H1N1) 2009 virus and compared it with that of other influenza A virus genotypes (see www.biol.sc.edu/~austin). In pandemic (H1N1) 2009 virus, synonymous (*π_S_*) and nonsynonymous (*π_N_*) nucleotide diversity (Technical Appendix) was significantly greater in NA than in HA (Table 1). In pandemic (H1N1) 2009 virus, *π_S_* in NA was >100× that in HA, and *π_N_* in NA was >50× times that in HA (Table 1). By contrast, in pre-2009 influenza virus subtype H1N1, *π_S_* and *π_N_* were similar in HA and NA (Table 1). Likewise, in influenza virus subtypes H3N2 and H5N1, *π_S_* and *π_N_* were similar in HA and NA (Table 1). Thus, pandemic (H1N1) 2009 virus was unique among serotypes in showing a marked difference in sequence diversity between HA and NA.

**Table 1:** Synonymous and nonsynonymous nucleotide diversity in hemagglutinin and neuraminidase genes of influenza A virus genotypes*

Genotype	HA				NA		
	No. sequences	*π_S_* ± SE	*π_N_* ± SE		No. sequences	*π_S_* ± SE	*π_N_* ± SE
Pandemic H1N1 (2009)	397	0.0041 ± 0.0015	0.0012 ± 0.0003		171	0.4626 ± 0.0493†	0.0616 ± 0.0065†
H1N1 (pre-2009)	105	0.0926 ± 0.0063	0.0171 ± 0.0017		105	0.0842 ± 0.0088	0.0126 ± 0.0016
H3N2	562	0.1178 ± 0.0094	0.0229 ± 0.0028		357	0.0871 ± 0.0077	0.0213 ± 0.0020
H5N1	109	0.0918 ± 0.0080	0.0189 ± 0.0026		116	0.1034 ± 0.0082	0.0194 ± 0.0027

*HA, hemagglutinin; NA, neuraminidase*; π_S_*, synonymous nucleotide diversity; *π_N,_* nonsynonymous nucleotide diversity. There was a significant difference (p<0.001) between *π_S_* and *π_N_* in all cases except HA of pandemic (H1N1) 2009. †Significant difference between *π_S_* or *π_N_* in NA and corresponding value in HA (z-test; p<0.001).

To test whether the difference between HA and NA in pandemic (H1N1) 2009 virus resulted from sampling error, we applied the same analysis to 92 epidemiologically matched pairs of HA and NA sequences from pandemic (H1N1) 2009 virus (see www.biol.sc.edu/~austin) collected in the same month (the same date, when possible) and from the same state (or the same country if not of US origin). *π_S_* was significantly greater in NA (mean ± SE 0.2537 ± 0.0183) than in HA (0.0030 ± 0.0011; p<0.001 by z-test). Likewise, in epidemiologically matched pairs, *π_N_* was significantly greater in NA (0.0215 ± 0.0022) than in HA (0.0012 ± 0.0003; p<0.001 by z-test).

In HA and NA genes of serotypes of influenza subtypes H1N1 (pre-2009), H3N2, and H5N1, *π_S_* was significantly greater than *π_N_* (Table 1). For pandemic (H1N1) 2009, *π_S_* was significantly greater than *π_N_* in NA (Table 1); *π_S_* was also greater than *π_N_* in HA, but the difference was not significant because diversity was low at synonymous and nonsynonymous sites (Table 1). *π_S_* was significantly greater than *π_N_* for each of the other 6 genes (Technical Appendix Table). A pattern of *π_S_* greater than *π_N_* indicates past purifying selection that has eliminated deleterious nonsynonymous mutations (*10*).

To obtain evidence regarding slightly deleterious variants subject to ongoing purifying selection (*11**–**13*), we examined gene diversity at synonymous and nonsynonymous polymorphic single-nucleotide polymorphism (SNP) sites in HA and NA genes (Table 2). In the NA genes of pandemic (H1N1) 2009 virus, subtypes H1N1 (pre-2009), H3N2, and H5N1, the gene diversity at nonsynonymous SNP sites was significantly lower than that at synonymous SNP sites (Table 2). The same pattern was seen in SNP sites in the HA gene of all serotypes except pandemic (H1N1) 2009 virus. Thus, the HA gene of pandemic (H1N1) 2009 virus showed a unique pattern in the absence of evidence of ongoing purifying selection decreasing the frequency of slightly deleterious variants.

**Table 2:** Mean ± SE gene diversity at synonymous and nonsynonymous polymorphic nucleotide sites in hemagglutinin and neuraminidase genes of influenza A virus serotypes*

Genotype	HA			NA	
	Synonymous	Nonsynonymous		Synonymous	Nonsynonymous
Pandemic H1N1 (2009)	0.0120 ± 0.0007 [173]	0.0112 ± 0.0006 [839]		0.2535 ± 0.0173 [179]	0.0863 ± 0.0060 [706]†
H1N1 (pre-2009)	0.0798 ± 0.0063 [198]	0.0506 ± 0.0019 [814]†		0.0765 ± 0.0083 [152]	0.0453 ± 0.0017 [712]†
H3N2	0.0710 ± 0.0086 [203]	0.0331 ± 0.0030 [793]†		0.0760 ± 0.0087 [177]	0.0332 ± 0.0029 [688]†
H5N1	0.1195 ± 0.0106 [184]	0.0552 ± 0.0027 [834]†		0.1120 ± 0.0098 [157]	0.0482 ± 0.0024 [645]†

*HA, hemagglutinin; NA, neuraminidase. Numbers of polymorphic nucleotide sites are indicated in brackets. †Gene diversity at nonsynonymous sites significantly different from that at synonymous sites (p<0.001; randomization test).

At 9 aa positions in HA, a residue not seen in our sample of pre-2009 influenza (H1N1) virus was fixed (100% frequency) in our sample of pandemic (H1N1) 2009 virus (Figure). The following amino acid replacements were involved; residue(s) in pre-2009 influenza (H1N1) are listed first: F/I/L88S, N101S, T256K, N/S275E, A/D/G277N, Q382L, G/R391E, F454Y, and S510A. Of these positions, 4 (88, 101, 275, and 391) were among those listed as having unique amino acid residues in pandemic (H1N1) 2009 virus on the basis of a smaller sequence sample by Ding et al. (*9*).

**Figure:**
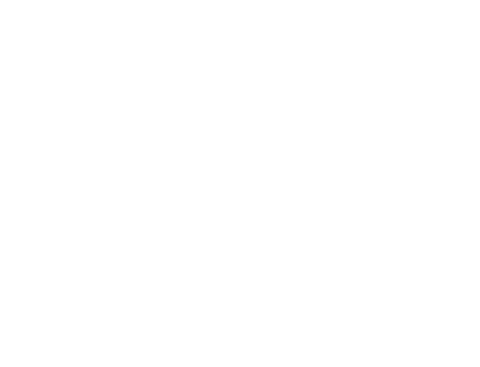
Structure of the pandemic (H1N1) 2009 virus hemagglutinin homotrimer, indicating (in red) the 9 aa positions in hemagglutinin at which a residue not found in pre-2009 influenza (H1N1) was fixed (100% frequency) in pandemic (H1N1) 2009 virus.

## Conclusions

Analysis of nucleotide sequences of HA and NA from 4 serotypes of influenza A virus showed a unique pattern of polymorphism in pandemic (H1N1) 2009 virus. In other serotypes, diversity of synonymous and nonsynonymous nucleotides was similar in HA and NA; in pandemic (H1N1) 2009 virus, HA showed much lower nucleotide diversity at synonymous and nonsynonymous sites than did NA. Of all serotypes analyzed, NA showed evidence of past and ongoing purifying selection against deleterious nonsynonymous mutations, and HA showed evidence of past and ongoing purifying selection of all serotypes except pandemic (H1N1) 2009 virus. These unique features of HA of pandemic (H1N1) 2009 virus imply that it has a more recent common ancestor than NA of the same serotype and that it has spread rapidly by frequent reassortment into a background of a much more ancient NA genotype.

The recent spread of HA of pandemic (H1N1) 2009 virus implies multiple events of reassortment, creating a population of viruses with an ancient and diverse NA gene and a much less diverse HA gene. The polymerase basic protein 1 gene also showed low diversity (Technical Appendix Table ), suggesting similar reassortment. Other genes of pandemic (H1N1) 2009 virus showed a level of diversity intermediate between that of HA and NA, suggesting that their association with this ancient and diverse NA may have resulted from earlier reassortment events. The bottleneck in the history of HA of pandemic (H1N1) 2009 virus explains the low genetic diversity and the absence of evidence of ongoing purifying selection because purifying selection is most effective when the population is large (*11**–**13*). Absence of ongoing purifying selection is thus consistent with a recent population expansion, of which pandemic (H1N1) 2009 virus shows evidence (*14*).

One factor that might have favored the spread of a recently evolved HA segment in the pandemic (H1N1) 2009 virus population would be the occurrence of >1 selectively favored aa replacements, causing a selective sweep (*15*) and reducing diversity at the HA locus. Such replacements in the ancestor of pandemic (H1N1) 2009 virus would likely be conserved in the pandemic (H1N1) 2009 virus population. The 9 aa residues in HA not found in our sample of pre-2009 influenza (H1N1), but fixed in our sample of pandemic (H1N1) 2009 virus, are candidates for selectively favored amino acid replacements in pandemic (H1N1) 2009 virus. Low diversity in >1 genes may be a recurring feature of newly emerged influenza A pandemics, supporting the need for vaccine development early in a pandemic to minimize mutation accumulation in viral genes of low initial variability.

## Supplementary Material

Technical Appendix Supplementary and Statistical, Methods.
