# Correction: Vol. 16, No. 11

**DOI:** 10.3201/eid1711.C11711

**Published:** 2011-11

In the article Reassortment of Ancient Neuraminidase and Recent Hemagglutinin in Pandemic (H1N1) 2009 Virus (P. Bhoumik, A.L. Hughes; http://wwwnc.cdc.gov/eid/article/16/11/10-0361_article.htm), errors were made in selection of the hemagglutinin (HA) and neuraminidase (NA) sequences for the initial and subsequent data sets. As a result, the authors incorrectly concluded that the NA gene of the pandemic (H1N1) 2009 virus is of a more ancient lineage than the HA. Other researchers (and the authors) have not been able to reproduce the findings when using HA and NA matched pairs from viruses chosen on the basis of geography and time and correctly have pointed out errors in the data set that make the original conclusions invalid.

Submitted by Priyasma Bhoumik and Austin L. Hughes

