# Supplementary material for: Reassortment of Ancient Neuraminidase and Recent Hemagglutinin in Pandemic (H1N1) 2009 Virus
Source: Emerg Infect Dis. 2010 Nov;16(11):1748–50. doi: 10.3201/eid1611.100361 (PMC3294508; doi:10.3201/eid1611.100361)
Supplement: Technical Appendix — Supplementary and Statistical, Methods. [file 10-0361-Techapp.pdf]

Article DOI: 10.3201/eid1611.100361

Reassortment of Ancient Neuraminidase and Recent Hemagglutinin in Pandemic (H1N1) 2009 Virus

Technical Appendix

Supplementary Methods

Sequences

We downloaded the coding sequences from 4 serotypes of influenza virus—H5N1, H3N2, H1N1 (pre-2009) and H1N1 (2009) from the NCBI Influenza Virus Resource (www.ncbi.nlm.nih.gov/genomes/FLU/Database/select.cgi?go=1). In the case of H1N1 (2009), we used only sequences deposited between January and July 2009 to obtain a representative picture of worldwide diversity at the start of the pandemic. The NA sequences from H1N1 (2009) were isolated in 23 different countries (Australia, Brazil, Canada, Chile, China, Denmark, Finland, France, Germany, Italy, Japan, Korea, Luxembourg, Mexico, the Netherlands, New Zealand, Norway, Philippines, Russia, Sweden, Thailand, United Kingdom, and the United States; the sequences from the United States represented 45 states. The NA sequences from H1N1 (2009) used from this study were isolated in 16 different countries (Brazil, Canada, China Columbia, France, Germany, Italy, Japan, Kazakhstan, Korea, Mexico, Nicaragua, Russia, Sweden, Thailand, and the United States; the sequences from the United States represented 36 different states. Pairs of epidemiologically matched HA and NA sequences (N = 92) were chosen to represent the same US state (or the same country in the case of non-US sequences) as close as possible to the same date (see www.biol.sc.edu/~austin). In 40 of 92 cases (43.5%), the paired sequences were from the same date; 65 of 92 (70.7%) were within 1 week of each other; and in no case were the two as much as 4 weeks apart. When more than one sequence was available for a given date and location, one sequence was selected at random for use in the matched pairs.

Statistical Methods

The sequences (Supplementary Tables 1 and 2, www.biol.sc.edu/~austin) were translated and aligned using the Prank program (*1*), and the alignment was imposed on the DNA sequences. Sequences containing undetermined nucleotides, premature stop codons and /or gaps were excluded from the analysis; partial sequences and laboratory strain sequences were also excluded from the analysis. Using the MEGA 4.0 program (*2*), we calculated the number of synonymous substitutions per synonymous site (d_S) and the number of nonsynonymous substitutions per nonsynonymous site (d_N) by the method of Li (*3*). This method was used because it takes into account the effect of transitional bias, which is particularly important in the case of 2-fold degenerate sites (*3*). The synonymous nucleotide diversity (symbolized π_S) is defined as the mean of d_S for all pairwise comparisons among a set of sequences, while the nonsynonymous nucleotide diversity (symbolized π_N) is the mean of d_N for all pairwise comparisons among a set of sequences. Standard errors of π_S and π_N were estimated by the bootstrap method (*4*).

In each of the viruses, gene diversity ("heterozygosity") was estimated at each polymorphic site by the formula:

$$1 - \sum_{i=1}^{n} x_i^2$$

where n is the number of alleles and x_i is the frequency of the ith allele in the set of sample sequences analyzed (*5,* p. 177). In coding regions, single-nucleotide polymorphisms (SNPs) were classified either as synonymous or nonsynonymous depending on their effect of the encoded amino acid sequence. Ambiguous sites were excluded from these analyses. The latter included sites at which both synonymous and nonsynonymous variants occurred in the set of sequences analyzed. Also excluded were certain polymorphic sites within codons with two or more polymorphic sites, when the polymorphism could be considered synonymous or nonsynonymous depending on the pathway taken by evolution. (For example, consider the two codons CTA and TTT. A mutation C→T in the first position would be synonymous if there were A in the third position, but not if there were T in the third position). Comparing gene diversities at synonymous and nonsynonymous polymorphic site provides evidence of ongoing purifying selection against slightly deleterious variants present in a population, since purifying selection will reduce the

frequency of slightly deleterious nonsynonymous variants in comparison to synonymous variants in the same genes (*6–9*).

Gene diversities were not normally distributed. Therefore, in testing for differences in mean gene diversity between synonymous and nonsynonymous SNP sites, randomization tests were used. In each test, 1,000 pseudo-datasets were created by sampling (with replacement) from the data; a difference between two categories was considered significant at the α level if it was greater than the absolute value of 100(1-α) % of the differences observed between the same categories in the pseudo-datasets.

The amino acid positions in HA at which a residue not seen in our sample of H1N1 (pre-2009) was fixed (i.e., at 100% frequency) in our sample of H1N1 (2009) were mapped on the crystal structure of HA from H1N1 (2009) (*10*) using RasTop version 2.2 (www.geneinfinity.org/rastop/).

References

1. Löytynoja A, Goldman N. An algorithm for progressive multiple alignment of sequences with insertions. Proc Natl Acad Sci U S A. 2005;102:10557–62. PubMed DOI: 10.1073/pnas.0409137102

2. Tamura K, Dudley J, Nei M, Kumar S. MEGA4: Molecular Evolutionary Genetics Analysis (MEGA) software version 4.0. Mol Biol Evol. 2007;24:1596–9. PubMed DOI: 10.1093/molbev/msm092

3. Li W-H. Unbiased estimates of the rates of synonymous and nonsynonymous substitution. J Mol Evol. 1993;36:96–9. PubMed DOI: 10.1007/BF02407308

4. Nei M, Kumar S. Molecular evolution and phylogenetics. New York: Oxford University Press; 2000.

5. Nei M. Molecular evolutionary genetics. New York: Columbia University Press; 1987.

6. Hughes AL. Micro-scale signature of purifying selection in Marburg virus genomes. Gene. 2007;392:266–72. PubMed DOI: 10.1016/j.gene.2006.12.038

7. Hughes AL. Near neutrality: leading edge of the neutral theory of molecular evolution. Ann N Y Acad Sci. 2008;1133:162–79. PubMed DOI: 10.1196/annals.1438.001

8. Hughes AL. Small effective population sizes and rare nonsynonymous variants in potyviruses. Virology. 2009;393:127–34. PubMed DOI: 10.1016/j.virol.2009.07.016

9. Hughes AL, Packer B, Welsch R, Bergen AW, Chanock SJ, Yeager M. Widespread purifying selection at polymorphic sites in human protein-coding loci. Proc Natl Acad Sci U S A. 2003;100:15754–7. PubMed DOI: 10.1073/pnas.2536718100

10. Xu R, Ekiert DC, Krause JC, Hai R, Crowe JE, Wilson IA. Structural basis of preexisting immunity to the 2009 H1N1 pandemic influenza virus. Science. 2010; 328:357–60.

Table. Synonymous and nonsynonymous nucleotide diversity in 6 genes of pandemic (H1N1) 2009 virus*

Gene	No. sequences	π_S ± S.E.	π_N ± S.E.
PB1	325	0.0017 ± 0.0003	0.0004 ± 0.0001†
PB2	197	0.0902 ± 0.0070‡	0.0052 ± 0.0005‡
PA	171	0.0884 ± 0.0009‡	0.0044 ± 0.0004‡
NP	103	0.0946 ± 0.0080‡	0.0094 ± 0.0012‡
NS1	129	0.0957 ± 0.0131‡	0.0231 ± 0.0028‡
M1	50	0.2030 ± 0.0269‡	0.0171 ± 0.0037‡

*Tests of the hypothesis that π_S or π_N equals the corresponding value in hemagglutinin (contact A.L.H. for Supplementary Table 1). There was a significant difference between π_S and π_N values and the corresponding values for neruaminidase (contact A.L.H. for Supplementary Table 1; p<0.001) in every case. There was a significant difference (p<0.001) between π_S and π_N in every gene. $\pi_{S,}$ *synonymous;* $\pi_{N,}$ nonsynonymous; PB, polybasic protein; PA, polyacidic protein; NP, nucleocapsid protein; NS, nonstructural protein; M, matrix.
†p<0.05.
‡p<0.001.
